# Evolution of Didacticians’ meta-didactical Praxeologies and Documentation Work

**DOI:** 10.1007/s10763-023-10367-w

**Published:** 2023-04-12

**Authors:** Gabriella Pocalana, Ornella Robutti

**Affiliations:** Mathematics Department “G. Peano”, University of Turin, Turin, Italy

**Keywords:** Documentation work, Inquiry communities, Meta-didactical praxeologies, Teacher professional development

## Abstract

This study is aimed to understand the connections between didacticians’ meta-didactical praxeologies for the design and implementation of a teacher professional development program and their documentation work for the program itself. Didacticians are mathematics education researchers with the role of teacher educators. Data presents a case study, in which two didacticians (the authors) generate documents for the work with seventeen in-service mathematics teachers working at the lower secondary level, on inquiry mathematics tasks. The results reveal relationships, not yet fully addressed in research, between the intertwining evolution processes of the didacticians’ meta-didactical praxeologies and their documentation work, nurtured by the collaboration with the teachers. The whole process is led by the evolving goal of the professional development program, from the promotion of the classroom implementation of inquiry mathematics tasks to the broader goal of building an inquiry community with the teachers. This study could contribute to the introduction of an interpretative model for the didacticians’ work for the design and implementation of a teacher professional development program, based on the combination of Meta-Didactical Transposition and Documentational Approach to Didactics frameworks.

## Introduction

During the last few years, a growing interest has been devoted to the study of teacher educators (e.g. Even, 2008; Karsenty, 2020; Loughran, 2014; Triantafillou et al., 2021), whose role in teacher professional development (PD) programs is highly complex and multifaceted. In this paper, we focus on the work of two mathematics education researchers (the authors), who are educators in a PD program for in-service mathematics teachers. This feature is in line with the Italian tradition in educational research (Arzarello & Bartolini Bussi, 1998), which sees academics directly leading PD programs for pre-service and in-service mathematics teachers. From now on, we use the term didacticians (Jaworski, 2008; Jaworski & Potari, 2021) to take into account their double role.

Our study addresses the issue, not yet fully explored in literature, of analyzing the relationship between didacticians’ meta-didactical praxeologies (Arzarello et al., 2014; Robutti, 2020) for the design and implementation of a teacher PD program and their documentation work (Gueudet & Trouche, 2009, 2012), based on the goals they set for the PD program. In the case study presented in this paper, didacticians’ meta-didactical praxeologies and their documentation work evolve, following the evolution of the goal set for the PD program in different phases. In the first phase, indeed, the didacticians’ goal for teachers’ PD was promoting the classroom implementation of inquiry mathematics tasks. So, the didacticians used to provide teachers with inquiry mathematics tasks (Laursen & Rasmussen, 2019; Maaß & Artigue, 2013) already designed to be implemented in the classroom. In the second phase, their goal evolved and became more complex, incorporating the goal of the previous phase and broadening it towards the building of an inquiry community (Jaworski, 2008) with the teachers participating in the PD program. According to this goal, the didacticians provide teachers with ideas and hints for the design of inquiry mathematics tasks and ask teachers to complete the design for their students and implement the tasks in their classrooms.

We claim that the work made by the didacticians for the design and implementation of the PD program — tasks for teachers, setting of the activities during the meetings … — involves a specific type of documentation work, in which didacticians generate documents for teachers’ PD. While the literature on documentation work is abundant in reference to teachers generating documents for their didactical activity in the classroom (Gueudet & Trouche, 2009, 2012; Gueudet et al., 2018), the didacticians’ documentation work for a PD program is less studied.

Our study could contribute to the introduction of an interpretative model for the didacticians’ work for the design and implementation of a PD program, based on the combination (Prediger et al., 2008) of the meta-didactical transposition (MDT) and the documentational approach to didactics (DAD) frameworks. Our research question is: *How is the evolution of the didacticians’ meta-didactical praxeologies for the design and the implementation of a PD program connected with their documentation work?*

To answer this question, in the following sections we will show that the didacticians’ documentation work is based on a large number of resources of diverse nature, material and human (Adler, 2000; Brodie, 2020; Gueudet & Trouche, 2010), and we will explain how the choice and the utilization schemes of these resources are connected with the didacticians’ meta-didactical praxeologies. The results that we present contribute to the analysis of didacticians’ work, both in terms of the MDT (Arzarello et al., 2014; Robutti, 2020) and the DAD (Gueudet & Trouche, 2009, 2012), thus showing internal connections between the two frameworks which have not yet been explored in detail so far.

## Inquiry in Mathematics Education

Inquiry-based learning (IBL) can be situated within the broader frame of active learning and teaching (Laursen & Rasmussen, 2019). It encompasses students’ learning experiences that are rich and meaningful: centered on their ideas and requiring their mental engagement. This approach is nurtured by the inherently social nature of classrooms. In this context, the teacher’s role is “to structure opportunities for students to reflect, analyze, synthesize, and communicate, and to make use of student ideas to structure these opportunities in the moment; to ensure that all students have an equal chance to participate and grow” (Laursen & Rasmussen, 2019, p. 3). Inquiry-based teaching refers to the teachers’ side of inquiry-based learning, which is allowing students to do inquiry (Maaß & Artigue, 2013). This requires teachers to have skills that go beyond exposing content and explaining concepts. Thus, fostering the acquisition of these skills is the aim of the PD program we are discussing in this paper.

### Inquiry Communities

Jaworski (2006, 2008), inspired by Wenger’s (1998) theory of communities of practice, proposes her conceptualization of inquiry communities. It refers to didacticians and teachers working together to explore and develop mathematics learning and teaching in the classrooms. In Wenger’s theory, belonging to a community of practice involves engagement, imagination, and alignment. In particular, alignment means engaging in forms of practice and ways of being, in order to conform to expectations and to the “normal desirable state.” In an inquiry community, the “normal desirable state” is continuously challenged with a questioning attitude (Jaworski, 2008). This attitude is termed critical alignment (Jaworski, 2006) and it involves a recognition that bringing a critical attitude to alignment, that is questioning, exploring, and seeking alternatives, can develop and change the normal state. Jaworski’s view of inquiry communities is based on the concept of co-learning inquiry, which means people learning together through inquiry. Inquiry, in this context, is meant at different levels: in mathematics classes with the students but also between teachers and didacticians, exploring how to use inquiry-based tasks with students. The building of an inquiry community is necessarily long and complex: it requires didacticians and teachers to be involved in the design of teaching materials, with tasks for students, and in collective reflections about possible improvements in the teaching–learning of mathematics.

In this study, we describe the transition of our PD program from the first phase, aimed to promote the implementation of inquiry mathematics tasks in the teachers’ classrooms, to the second phase, which can be conceptualized as the building of an inquiry community among didacticians and teachers. At the beginning of the first phase, the didacticians involved the teachers in an inquiry cycle (plan, act and observe, reflect and analyze, feedback) in the design process, which led to a continuous process of reconceptualization and redesign of teaching materials, as described in Jaworski (2008).

The transition between the two phases implied an evolution of the meta-didactical praxeologies of both the communities involved in the PD program, as we describe in the following sections, focusing on the didacticians’ side of this complex phenomenon. Pursuing critical alignment between didacticians and teachers was the leading goal of this evolutionary path.

## Meta-didactical Transposition

We use the MDT framework (Arzarello et al., 2014; Robutti, 2020) as an interpretative lens for the phenomena we are studying. The MDT allows us to describe the interactions between two communities, teachers and researchers, who have the role of educators in a PD program. This description is dynamic, showing the evolution over time of their respective meta-didactical praxeologies.

The term praxeology was introduced by Chevallard (1999): it refers to the combination of a practical component, or *praxis*, and a theoretical component, or *logos*. In the case of didactical praxeologies, this combination is referred to teachers’ activity in the class. The practical component includes tasks to be solved and techniques to solve them, while the theoretical component includes the justification and the explanation of the techniques. In the MDT framework, the praxeologies involved are meta-didactical ones, because they are referred to teachers’ and didacticians’ activities in the context of a PD program, not to teachers’ activities in their classes. Different techniques can be used to engage in the same type of tasks and the justifications for this *praxis *can refer to different *logos*, depending on one’s institutional position. The MDT framework takes into account the reciprocal influences of the different communities and models the evolution of their meta-didactical praxeologies, describing it as the result of internalization processes of elements that were previously external to the communities.

Cusi et al. (2023) deepen some theoretical aspects of the MDT, which they call MDT.1 to distinguish it from the new version they introduce, named MDT.2. They present the integration of new elements to describe the internalization process. For instance, they integrate within the MDT.1 some elements of the boundary object (BO) perspective (Star, 2010) to investigate the ways in which teachers and researchers work collaboratively on the same object. BOs, following Star (2010), are “a sort of arrangement that allows different groups to work together without consensus” (p. 602). The work on a BO, according to Cusi et al. (2023), represents “the driving force that triggered the evolution of both teachers’ and researchers’ praxeologies.” Inspired by their integration work, in this paper, we aim to introduce new elements to describe the didacticians’ meta-didactical praxeologies, explaining how they are connected with their documentation work in its evolution over time.

## Documentational Approach to Didactics

The DAD framework (Gueudet & Trouche, 2009, 2010, 2012; Gueudet et al., 2012; Trouche et al., 2019) has been introduced to study teachers using different kinds of resources to prepare their lessons and to support students’ learning. The DAD is inspired by the instrumental approach (Rabardel, 2002), which introduced the distinction between an artifact and an instrument. The DAD introduces a parallel distinction between resources and documents: a document consists of a set of resources and related utilization schemes for a particular class of situations. The process by which documents are generated is named documentational genesis (Gueudet & Trouche, 2009) and implies the active role of the subject.

The relational formula, representing the process of documentational genesis (Gueudet & Trouche, 2009) is: “Document = Resources + Utilization schemes.” Utilization schemes are constituted by: classes of situations (in which resources are used), rules of action (stable elements in the way the resources are used), and operational invariants (which are part of the set of beliefs and knowledge of the teacher and are both driving forces and outcomes of the teacher’s activity). Documents comprehend both a material and a psychological component, like the instrument in the sense of Rabardel.

Usually, scholars rely on the DAD to understand teachers’ work and growth via understanding changes in their documentation work, which Gueudet and Trouche (2009) describe as “looking for resources, selecting/designing mathematical tasks, planning their succession and the associated time management, etc.” (p. 199). Gueudet et al. (2012), however, observed the documentational genesis of trainee teacher educators, in an experiment with two teams of “training path designers,” who had to become teacher educators using the path designed by another team. The “training paths” were considered, in that study, to be resources for the trainee teacher educators who decided to use them for setting up their own training. In this paper, we aim to do something slightly different: to adopt the documentational approach for the study of the work of teacher educators, who are also researchers (didacticians), when they use different kinds of resources to design all the documents for the work with the teachers. As in the case of teachers, also for the didacticians resources can be of many different kinds, material and human (Adler, 2000; Brodie, 2020; Gueudet & Trouche, 2010). For example, we can acknowledge as resources: literature in the field of mathematics education, discussions with colleagues, written and oral interactions with the teachers, written protocols produced by the teachers during the meetings, issues raised by the teachers during the collective discussions, and many others.

In the PD program object of our study (Pocalana et al., 2023a, b), the didacticians’ documents are specific for teacher PD and could constitute resources for teachers (we use this expression to distinguish them from the resources for the didacticians), for the generation of documents for the work in their classrooms, in a cyclic process. This characteristic represents a difference from the case presented, for example, by Kieran et al. (2013), about the researchers’ documentational genesis. In their study, in fact, the DAD is applied to documents designed by researchers directly for the students and not for the work with teachers.

Psycharis and Kalogeria (2018) provided an analysis of the documentation work of trainee teacher educators, who have to practice designing resources for teachers during a PD program. In their case, like in our study, the focus of the research was the documentation work of the educators for the work with teachers. The subjects, although, were trainee educators, that is secondary school mathematics teachers, who were facing the transition towards the role of a teacher educator, under the guidance of academic trainers. In our case, instead, educators are themselves academics, not involved in their own professional development path, so not having trainers providing them with resources for their work.

## Methodology

### Characteristics of the PD Program

Our study is focused on the transition of a teacher PD program from its first phase, which lasted 3 years, to its second phase, still ongoing. The didacticians’ goal, in the first phase, was to create a theoretical and practical common ground between the two communities, based on the principles of the IBL. The main IBL principles taken into account were the following: the proposal of a student-centered way of learning and teaching, in which students learn to inquire and are introduced to mathematical and scientific ways of inquiry (Maaß & Artigue, 2013; Swidan et al., 2023); the building of a classroom context in which “students raise questions, explore situations, and develop their own ways towards solutions” (Maaß & Artigue, 2013, p. 780). The didacticians provided teachers with theoretical references and promoted the implementation of inquiry mathematics tasks in their classrooms, providing them with ready-made tasks, one for each meeting.

The second phase marked an evolution in the goal of the collaboration between didacticians and teachers, towards the building of an inquiry community in which teachers and didacticians collaboratively design tasks for students (Table 1 shows the main characteristics of the two phases of the PD program). This involved an evolution of the meta-didactical praxeologies of both communities: teachers had to be active participants in the design of tasks for their students (one per meeting) and didacticians had to leave space of freedom for teachers’ design choices. Teachers’ meta-didactical praxeologies could be the object of future studies, while this study takes into consideration the didacticians’ side of the evolution.

**Table 1:** Description of the two phases of the PD program (the main differences are underlined)

First phase of the PD program		Second phase of the PD program	
Didacticians	Teachers	Didacticians	Teachers
- Provide teachers with theoretical references on the IBL, before the presentation of the activities. - Provide teachers with a teacher-sheet, with questions for the teachers, and a student-sheet, containing an inquiry mathematics task for students, in a complete form. - Ask the teachers to solve the task as if they were students. - Orchestrate ex-post collective reflections with teachers, about the classroom implementation of the activities.	- Work in groups, during the meetings, to answer the didacticians’ questions. - Work individually, after the meetings, to generate teachers’ documents for their classroom work. - Are responsible, individually, for the classroom implementation of the activities. - Are involved in ex-post collective reflections with the didacticians, about their classroom implementations.	- Make reference to the IBL theoretical background, shared with the teachers during the previous years, and provide new “theory pills”, when necessary. - Provide teachers with an activity-sheet with ideas and hints for the design of an inquiry mathematics task for students and with questions for the teachers. - Engage teachers in a priori collective reflections, during the meetings, about the task design for students. - Ask the teachers to solve the task as if they were students. - Orchestrate ex-post reflections with teachers, both about their task design choices and about the classroom implementation of the activities.	- Are involved in a priori collective reflections with the didacticians, about the task design for students. - Complete the task design, generating teachers’ documents, and answer the didacticians’ questions, working in groups (without the didacticians), during and after the meetings. - Are responsible, individually, of the classroom implementations of the activities. - Are involved in ex-post reflections with the didacticians, both about their task design choices and about the classroom implementation of the activities.

### Methodological Approach

In this paper, we present an instrumental case study (Mills et al., 2010), which constitutes an opportunity to exemplify our methodological proposal. We aim to carry out an in-depth reading of the complex processes we are studying, combining (in the sense of networking, see Prediger et al., 2008) the MDT and the DAD frameworks. In our combined conceptualization, the MDT framework helps us to answer the question, formulated in the DAD terms: “Why didacticians generated documents with certain characteristics?” The DAD framework, on the other hand, helps us to answer the question, incorporating the MDT terminology, “How didacticians created documents for the work with teachers, as part of their meta-didactical praxeologies?”.

In our analysis, a didacticians’ meta-didactical praxeology is referred to a particular phase of the PD program, while the resources’ utilization schemes are referred to specific didacticians’ documents. The task of the didacticians’ meta-didactical praxeology of the first phase of the PD program is to provide teachers with inquiry mathematics tasks, ready to be implemented in their classrooms. In the second phase, the task evolves into fostering the building of an inquiry community with the teachers, in which the didacticians provide hints and ideas for the design of inquiry mathematics tasks, which has to be completed by the teachers. The techniques of a didacticians’ meta-didactical praxeology are the operative ways in which they accomplish the task, while the *logos* component constitutes the theoretical justification of their choices for the design and the implementation of the PD program. Our perspective in conducting the analysis is that the documents are generated by the didacticians as part of their techniques and reflect the *logos* component of their meta-didactical praxeologies. The class of situations of a document is the type of circumstances in which the document has to be used, related to a specific goal for the PD program. The rules of action constitute observable aspects of the operational invariants of the document that is the principles inspiring the design and guiding the utilization of the document. The operational invariants are, in our analytical hypothesis, strictly connected with the *logos* component of the didacticians’ meta-didactical praxeology for a certain phase, because the documents generated have to contribute to the didacticians’ goal for that phase.

The MDT framework (Arzarello et al., 2014) develops the concept of didactical praxeologies (Chevallard, 1999) to encompass meta-didactical praxeologies, referred to the meta-didactical activity occurring during a teacher PD program, led by educational researchers. In this study, we develop the concept of documentational genesis, coming from the DAD framework, including the generation of didacticians’ documents for the work during a teacher PD program. In a cyclical way, the didacticians’ documents constitute resources for teachers for the generation of teachers’ documents, to be used with students. These teachers’ documents can constitute, in turn, new resources for the generation of new didacticians’ documents which take into account teachers’ ideas, needs, and task design choices for their students.

We think that the results we obtained, answering our research question, could be generalizable to the study of diverse types of teacher PD programs, in different contexts, as a confirmation of the validity of our proposal of combination (Prediger et al., 2008) of two theoretical frameworks (MDT and DAD).

### The Participants in the PD Program

The PD program analyzed in our study (Pocalana et al., 2023a, b) is part of the Turin University project SSPM (Scuole Secondarie Potenziate in Matematica), which is connected with the Italian National project Liceo Matematico. The national project is mainly directed at upper secondary schools, but the Turin experience also involves primary and lower secondary schools. The aim of the project — carried out according to an institutional agreement between the schools and the university — is to provide additional mathematics classes to the students, taught by the mathematics teachers of their own schools, besides the curricular classes. To ensure that the additional classes become a real learning opportunity for students, the mathematics teachers involved in the project have to follow every year a PD program, held by educational researchers of the university department of reference. The additional mathematics classes are devoted to inquiry mathematics tasks in disciplinary or interdisciplinary STEAM contexts.

We focus on a PD program addressed to lower secondary school (grades 6–8) mathematics teachers, started in 2017. It consists of two phases, attended by the same teachers: the first lasted for the first 3 years, while the second started with the fourth year, in 2020, and it will last 3 years too. In both phases, the synchronous activity for every year consists of 10 meetings of 2 h each, while the asynchronous phase consists of 10 h of individual work on the Moodle platform and 33 h of classroom experimentation. We will focus, in particular, on the first year of the second phase, a year of transition and evolution for the meta-didactical praxeologies of both the communities involved. The participants are 17 lower secondary school mathematics teachers, who have already attended the first phase of the PD program and have been informed that the second phase would have asked them for something more: a qualitative leap in their modalities of participation in the PD program entailing their direct involvement into the design of inquiry mathematics tasks. Eight participants have a master’s degree in mathematics or physics, while 9 have a master’s degree in biology, natural sciences, or agriculture. The majority of them are women, there are only 3 men. They are all experienced teachers, only 2 of them have less than 10 years of service, 9 of them have more than 15 years of service, and 5 of them have more than 20 years.

### Data Collection

The data presented in this paper have been selected from a larger set of collected data. They derive from all the resources for teachers provided by the didacticians for the work during the first phase and the first year of the second phase of the PD program (the latter held online due to the COVID-19 restrictions). All these resources for teachers are retrievable on the web platform (Moodle) used for the asynchronous interactions between teachers and didacticians. In particular, we collected:

1. The sheets provided to teachers by the didacticians during the meetings
2. The slides projected by the didacticians during the meetings, to introduce the activities
3. The transcripts of the video recordings of the online meetings of the first year of the second phase. The video-recorded interactions occurred when teachers and didacticians were all together in the main session of the online meeting because in the separated sub-sessions, it was not possible to record, due to technical limitations
4. The teachers’ protocols, provided in response to the didacticians’ requests. They include, for example, the design of inquiry mathematics tasks for students, teachers’ answers to reflection questions, or reports of classroom experimentations

### Data Analysis

Among the data enlisted in the previous section (points 1 and 2), we chose examples that seemed significant to us because they exemplify the didacticians’ meta-didactical praxeology in a specific phase of the PD program. They are both considered material parts of documents generated by the didacticians for the work during the PD program and as resources for the didacticians’ documentation work related to other, subsequent, documents. For all the analyzed documents, we traced the main resources on which the didacticians’ relied for their generation and the utilization scheme, composed of the class of situations, the rules of action, and the operational invariants.

The DAD has developed a specific methodology, the reflective investigation of teachers’ documentation work, which gives a major role to teachers themselves. The active involvement of teachers, with a reflective stance, is needed because they have access to their documentation work and they can make visible some hidden resources (Gueudet & Trouche, 2012). We endorse the same principle and base our analysis on the reflections of the didacticians (the authors), scrutinizing their documentation work with an introspective attitude. The description of didacticians’ meta-didactical praxeologies is also based on their reflections on their practices and justifications for their practices, referred to as the design of the work in the PD program. The methodological issue of didacticians analyzing their own work has been already taken into account, for example, in the work of Berry (2008), who adopted a self-study approach (Hamilton, 1998), to obtain a deep understanding of her own practices. So, we analyze our data coherently with the reflective investigation and self-study principles. We described didacticians’ meta-didactical praxeologies for the first and the second phase of the PD program, identifying their practices and justifying discourses for the design and implementation of the PD program itself. After that, we identified the relationships between the components of the utilization scheme of the didacticians’ document and the *praxis* (task and technique) and the *logos* components of the didacticians’ meta-didactical praxeology, referred to the phase in which that particular document was generated. In the “Results” section, we always use the term praxeology to designate meta-didactical praxeology.

The data enlisted in points 3 and 4 of the previous section were analyzed following the principles of qualitative thematic analysis (Braun & Clarke, 2006), with an inductive approach. Our aim was to identify, among the emerged themes, elements that the didacticians took into consideration when they had to generate documents for the work with the teachers. These elements were categorized as resources that contributed to the didacticians’ documentation work. Initially, each one of us coded these data and, after comparing our work, we kept the common themes, merging or deleting some others. For example, two of the themes that emerged were as follows: “Some students need support to carry out the activity” and “Design of help cards.” Having identified the themes, we related them with the didacticians’ documents used during the course, to identify how they had contributed to their genesis. At every stage, the authors worked, at first, individually, and, in a second moment, they met together to share and discuss the results of their analysis.

## Results

In this section, we describe how the evolution of the didacticians’ praxeologies for the design and the implementation of the PD program, from the first to the second phase of the PD program, is connected with their documentation work. To do so, we present two examples of documents generated by the didacticians, one for each phase. For each example, we explain how it is connected with the didacticians’ praxeology of the respective phase. All the sheets provided to teachers by didacticians, the discussions’ excerpts, and the teachers’ protocols are translated from Italian by the authors. The names of the teachers are all pseudonyms.

### First Phase of the PD Program

In the first phase of the PD program, the didacticians aimed to promote the implementation of inquiry mathematics tasks in the teachers’ classrooms, presenting a basic theoretical background from the literature on the IBL and providing teachers with sheets with inquiry mathematics tasks ready to be proposed in their classrooms. Besides that, the didacticians asked teachers to answer questions about the didactic value and collocation of the tasks. In light of this, their praxeology in the first phase can be described as follows (Table 2):

**Table 2:** Description of didacticians’ praxeology for the design and implementation of the PD program in the first phase

Task	Promoting the classroom implementation of inquiry mathematics tasks
Technique	• Selecting and organizing a basic theoretical background on the IBL • Providing teachers with a student-sheet with an inquiry mathematics task, ready to be presented in their classrooms • Providing teachers with a teacher-sheet, with questions about the prerequisites that students must have to solve the task, the didactical value of the task, and the connections they see between the task, the curriculum, and their programs
Logos	Literature on the IBL (Laursen & Rasmussen, 2019; Maaß & Artigue, 2013)

Based on the praxeology described above, the didacticians carried out a documentation work coherent with their goal for the PD program.

#### Documentation Work in the First Phase

**Fig. 1:**
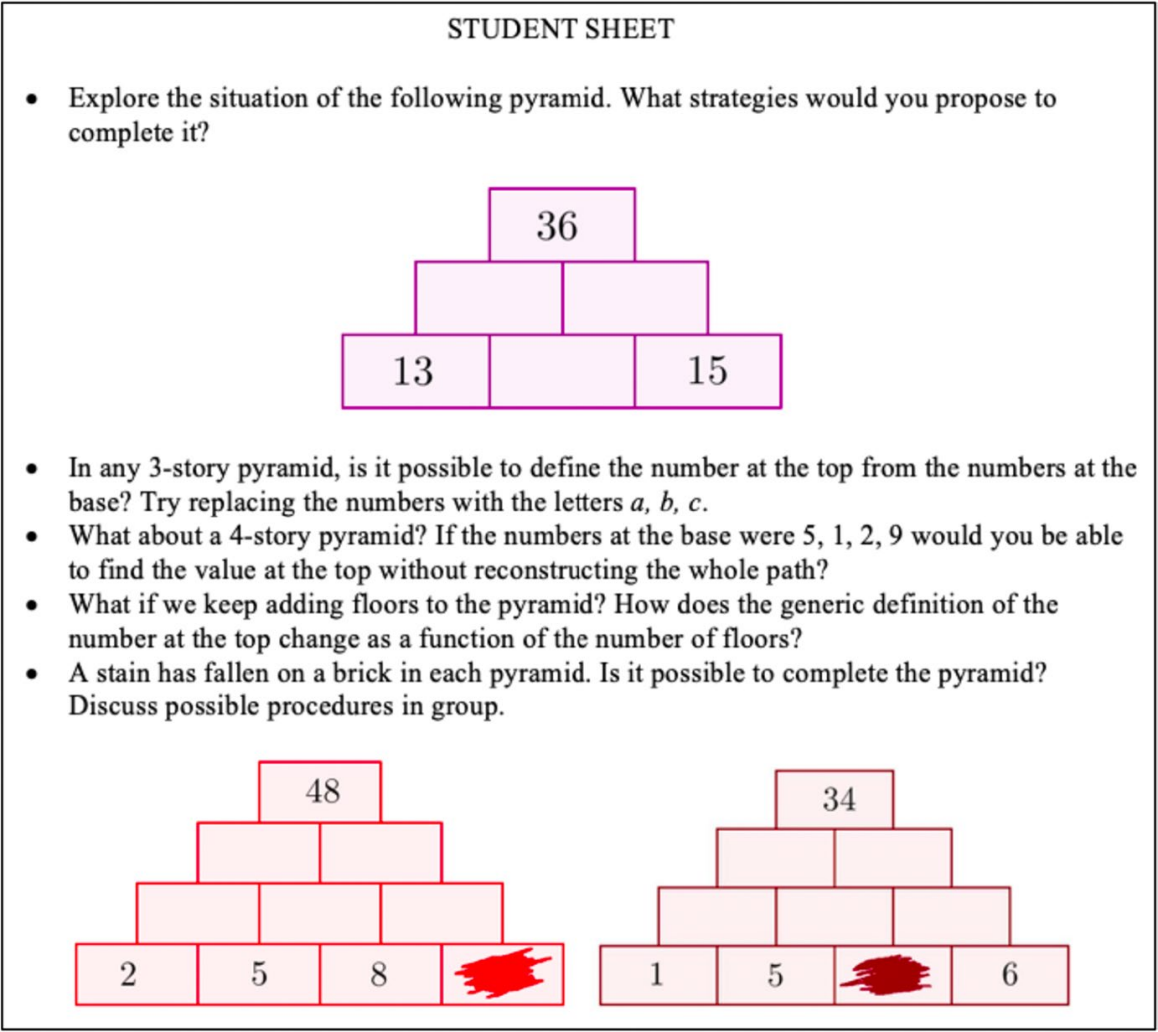
Example of a student-sheet provided to teachers by the didacticians during the first phase

**Fig. 2:**
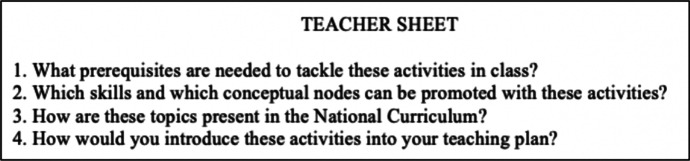
Example of a teacher*-*sheet provided to teachers by the didacticians during the first phase

During the first phase of the PD program, the didacticians generated documents for the meetings with the teachers with specific features, which remained almost stable throughout the 3 years. In the following, we present an example of the student-sheet (Fig. 1) and of the teacher-sheet (Fig. 2), which represented resources (Maracci et al., 2023) for teachers, provided by the didacticians during every meeting.

The main resources on which the didacticians drew upon for their documentation work are both materials and humans. Among the material resources, we can list the literature on the IBL (e.g. Laursen & Rasmussen, 2019; Maaß & Artigue, 2013), from which the didacticians derived the theoretical basis for the task design and the proposal of the activity for students. Furthermore, the didacticians were inspired by the Italian ArAl project (Malara & Navarra, 2018), based on the theoretical framework of early algebra. This framework aims to demonstrate how it is possible and effective to start introducing algebraic thinking much earlier than the end of lower secondary school, as usually happens. Among the activities proposed within the project, there is an entire section dedicated to the pyramids of numbers (Navarra & Giacomin, 2003), which constituted the inspiration for the inquiry mathematics task proposed in the student-sheet (Fig. 1). Among the human resources, we can indicate the discussions that occurred between the group of the didacticians and colleagues holding other PD programs related to the same SSPM project.

Based on the diverse resources listed above, the didacticians developed a utilization scheme (Table 3) for the document for the meeting, in which they provided teachers with the student-sheet (Fig. 1) and the teacher-sheet (Fig. 2). We can see that there is a strong relationship between the rules of action of the utilization scheme described in Table 3 and the technique of the didacticians’ praxeology described in Table 2, as well as between the operational invariants and the *logos* of the praxeology. In fact, the didacticians’ documentation work is part of their praxeology relative to the specific phase of the PD program in which the document is generated.

**Table 3:** Utilization scheme of a document of the first phase

Class of situations	Work with the teachers on the IBL approach, during the meetings of the first phase of the PD program
Rules of action	• The student-sheet, containing a task based on the principles of the IBL approach, is ready to be presented in the classroom • The teacher-sheet contains questions regarding the effective inclusion of the task in their didactic planning
Operational invariants	• It is important that teachers have a collection of inquiry-based tasks to draw upon for their classroom work • It is important that teachers see inquiry-based tasks inserted in their didactic planning and not as a sporadic diversion from their usual work in the classroom

### Second Phase of the PD Program

In the second phase of the PD program, the didacticians aimed to build an inquiry community with the teachers, promoting a questioning attitude, that is exploring, and seeking alternatives to the “normal” practice. They started to engage the teachers directly in task design, in collective discussions about their task design choices and about their classroom implementations, in an inquiry cycle (Jaworski, 2008). Didacticians’ praxeology for the second phase of the PD program is described in Table 4.

**Table 4:** Description of didacticians’ praxeology in the second phase

Task	Fostering the building of an inquiry community with the teachers
Technique	• Providing teachers with an activity-sheet, with hints for the design of inquiry mathematics tasks for their students • Asking teachers to complete the task design and to answer questions about the justifications of their task design proposal • Involving teachers in collective discussions on their task design and classroom implementations
Logos	Literature on the IBL (Laursen & Rasmussen, 2019; Maaß & Artigue, 2013), and on the Inquiry Communities (Jaworski, 2006, 2008)

The technique is constituted by elements that were not present in the previous phase, mainly teachers’ engagement in task design, in a collective work (Gueudet & Trouche, 2012), and the request to justify their design choices. Didacticians provided teachers with ideas and hints for inquiry mathematics tasks, following the principles of the IBL approach, in continuity with the previous phase of the PD program. The tasks, however, were presented in a not completed formulation and teachers had to complete their design to generate teaching materials.

We will see in the following how this praxeology finds a correspondence in the didacticians’ documentation work, carried out in that period.

#### Documentation Work in the Second Phase of the PD Program

**Fig. 3:**
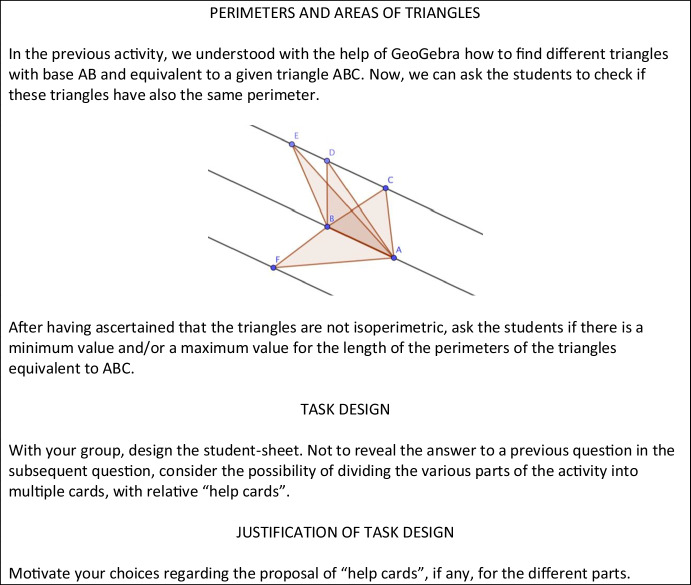
Example of activity-sheet which was provided to teachers during the 8th meeting of the second phase

In each meeting of the second phase, the didacticians provided teachers with only one sheet, named activity-sheet, composed of two sections: the first one with ideas and hints for the design of a mathematics inquiry-based task for students and the second one devoted to meta-didactical reflections. In the activity sheet (Fig. 3) of the 8th meeting of the second phase, we can detect a shared praxeological element, referred to the task design for students, co-constructed among teachers and didacticians, during their joint work. This element is constituted by “help cards” with single suggestions, to be provided, in a flexible way, to the students, or the groups of students, who need them at a specific point of the activity. “Help cards” are thought to be provided when needed, besides the usual student-sheet, which is the same for everyone. They are inspired by the literature in mathematics education, in particular by Cusi et al. (2017), which constituted a resource for this document, as we will describe in the following. The introduction of this element is motivated by the need to design inquiry-based activities for the wall class, in an inclusive way, in order to address the issue, raised by the teachers, that some students cannot carry out these kinds of activities without support. So, we can identify an internalization process, for both the communities of didacticians and teachers, of the “help cards” element in the design of teaching materials for students, which can be described also as a critical alignment process for the inquiry community that was being created during the PD program. Indeed, the idea of “help cards” was external to both communities and it was born from the joint work of didacticians and teachers during the meetings and from the reflections on teachers’ experimentations in their classrooms. The main resources on which the didacticians drew upon for their documentation work are, also in this case, both materials and humans.

##### Resource 1

This includes teacher-sheets and student-sheets, which were provided during the first phase of the PD program. Among the resources that contributed to the generation of this document, we first mention the teacher-sheets and the student-sheets used in the 3 years of the first phase of the PD program, because they influenced the design of the activity-sheets of the second phase. The structure, in fact, is not very dissimilar, because both phases of the PD program have been centered on IBL tasks. But it has some important new features: for example, in the activity-sheet, there are ideas for the students’ task, but not a completely designed task, ready to be presented in the classroom.

##### Resource 2

**Fig. 4:**
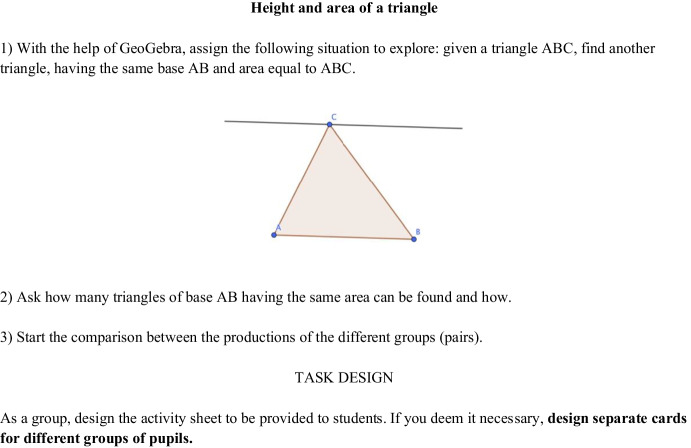
First part, with hints for the task for students, of the activity-sheet provided to teachers by the didacticians during the 7th meeting of the second phase

This includes activity-sheets, which were provided during the previous meetings of the second phase of the PD program. For the choice of the mathematics task, we can consider a resource the activity-sheet of the previous meeting (Fig. 4).

During the 8th meeting, the didacticians proposed a further exploration connected to the mathematics task of the 7th meeting, centered on the relationship between the base, height, area, and perimeter of a triangle. This choice is motivated by the assumption that, as stated in Laursen and Rasmussen (2019, p. 2–3), IBL is based on “a longer-term trajectory […]. These coherent task sequences scaffold students’ mathematical work on challenging problems over weeks of instruction.”

##### Resource 3

This includes mathematics education literature. For the proposal of the task present in the activity-sheet, we list as a resource a contribution to the field of mathematics education literature, made by Arzarello et al. (2002). This work discusses the significant role of dragging in supporting the cognitive shift from the perceptual level to the theoretical one and back in students’ mathematical activity. According to the authors, “dragging supports the production of conjectures: exploring drawings by moving them, looking at the ways after which their forms change (or do not change), allows users to discover their invariant properties” (p. 1). The didacticians, based on these results, during several meetings with the teachers, in both phases of the PD program, have been proposing tasks requiring dragging practices, to support the exploration activity of the students in the GeoGebra environment.

##### Resource 4

For the part of the activity-sheet related to the reflections on task design, we acknowledge as resources contributions in mathematics education literature, in particular the paper by Cusi et al. (2017), related to an experimentation connected to the European project FaSMEd. This experimentation involved teachers and researchers, working together to study the potentialities of digital tools to foster the activation of formative assessment strategies within the classroom. In this context, they described a methodology in which students were provided with three digital worksheets: a “problem worksheet,” introducing the mathematical task; a “helping worksheet,” aimed at supporting students who face difficulties with the “problem worksheet,” by making specific suggestions; and a “poll worksheet,” prompting a poll among proposed options. The didacticians, during the 7th meeting, proposed to teachers to study some characteristics of this project, to understand if there were elements suitable for the PD program situation they were encountering. In fact, for several months, they had been facing the teachers’ need to support the most fragile students during inquiry-based activities (Pocalana et al., 2023a). From the joint work of teachers and didacticians, possible adaptations of the elements of the FAsMEd project emerged, in particular regarding the subdivision of the “helping worksheet” into different “help cards,” to be provided flexibly to the students, based on their real difficulties, in a specific moment. Based on this new idea, the didacticians generate the document for the 8th meeting, asking teachers to integrate the new “help card” element in the design of their teaching materials for the classroom experimentations.

##### Resource 5

This includes collective discussions among teachers and didacticians. The collective discussions that occurred during the previous meetings were important resources for the generation of this document, because they testify to the emergence of a new idea: the “help cards,” based on the “helping worksheets” proposed in the FAsMEd project (Cusi et al., 2017). We will report, as an example, three excerpts from the collective discussion, which occurred during the 7th meeting, in which the two themes “Some students need support to carry out the activity” and “Design of help cards” emerge (italics from the authors).

Teresa: We have thought of *preparing “help sheets”* but without already addressing them to strong groups or weak groups, *we have designed them by points* [...]

Anna: *We give “help cards”* to those who ask them, so *we didn’t think of designing entire sheets*, we leave more depending on how the work in the groups goes.

Teresa: Yes, because we create homogeneous students’ groups, however, *we cannot a priori understand where they will get stuck, what their difficulty will be* and, therefore, *we are ready to give a specific hint, depending on what they ask for.*

##### Resource 6

This includes teachers’ protocols containing the design of tasks for their students, their answers to reflection questions, and their reports of classroom implementations.

The didacticians based their documentation work also on the teachers’ task design choices, the motivations they reported, and the issues they raised. This is coherent with the goal of the second phase of the PD program, of taking into account teachers’ feedback, reflections, and active involvement at all levels.

In the following excerpt, we can detect both the themes that already emerged during the collective discussions: “Some students need support to carry out the activity” and “Design of help cards” (italics from the authors):

Franca: We left the choice of how to solve the task: using scissors and cardboard to build material models or working with GeoGebra. This choice is due to the fact that *the activity has been thought to be proposed to the whole class and not all the students are good enough at using GeoGebra to be able to do this task on their own.* […] *We prepared a series of “help cards”,* which are not differentiated by level but follow the different stages of the activity and *can be offered to any group that needs them*.

The didacticians, therefore, decided to integrate the “help cards” element in the requests contained in the activity-sheet of the 8th meeting, because they recognized it as an internalized praxeological element, both for the teachers and the didacticians themselves.

We would list many other resources that contributed to the didacticians’ documentation work, as in the case of the first document described, but we chose to analyze in detail the most significant ones, in our eyes.

The utilization scheme of the document for the 8th meeting of the second phase is summarized in Table 5.

**Table 5:** Utilization scheme of a document of the second phase

Class of situations	Work with the teachers on the IBL approach, during the meetings of the second phase of the PD program
Rules of action	• To propose ideas for the mathematical task, in the first section of the activity-sheet, involving further explorations connected to the task of the previous meeting • To devolve the completion of the task design to the teachers • To ask teachers to design different “help cards” for students
Operational invariants	• It is important to connect the activities proposed in the classroom in the context of explorations which can last several weeks • Teachers should have a role as designers of teaching materials for their students, according to what emerged also in ICMI Study 22 (Watson & Ohtani, 2015) • It is important to take into account the need, expressed by the teachers, to help the students who cannot solve the tasks without support

We can notice, as for the first phase, a strong relationship between the rules of action of the utilization scheme (Table 5) of this document and the technique of the didacticians’ praxeology described in Table 4. The rules of action contain specific elements relative to the particular document for the 8th meeting, while the technique component is relative to the general praxeology of the second phase of the PD program. The operational invariants reflect the general *logos* of the same praxeology, but they are related to the specific context for which the document is thought.

## Discussion and Conclusion

This study aimed to address the issue of understanding how the evolution of the didacticians’ meta-didactical praxeologies for the design and implementation of a PD program is connected with their documentation work. In our case study, the results show that the evolution of the didacticians’ meta-didactical praxeologies from the first to the second phase of the PD program is reflected in the evolution of their documentation work. What emerged from the data analysis brought to the fore a complex intertwining of resources of diverse nature, both material and human, that contributed to the genesis of didacticians’ documents for the work with the teachers during the PD program.

In the first phase, the didacticians’ documentation work is an expression of their meta-didactical praxeology, whose task is promoting the classroom implementation of inquiry mathematics tasks. The technique, indeed, includes the provision by didacticians of teaching materials, based on the theory of the IBL approach (*logos* component), ready to be used by teachers with their students. In the second phase, the resources used and the utilization schemes of the didacticians’ documents testify to their choice of involving the teachers in the task design for their students. The analyzed documents, indeed, take into account teachers’ needs, their feedback, and the reasons for their design choices for students, coherently with the new task of the didacticians’ meta-didactical praxeology, which is fostering an inquiry community with the teachers. Critical alignment has been pursued by the didacticians basing the genesis of their documents on every possible source of information about teachers’ practices, motivations, and goals. In particular, the didacticians exploit as resources the teachers’ reports of classroom activities and the collective discussions during the meetings. We can call this kind of resources “human resources” (Adler, 2000; Brodie, 2020; Gueudet & Trouche, 2010), because they come from the interactions with the teachers and testify to the intention of the didacticians to involve them in a real exchange of ideas, proposals, and purposes for the co-work during the PD program.

**Fig. 5:**
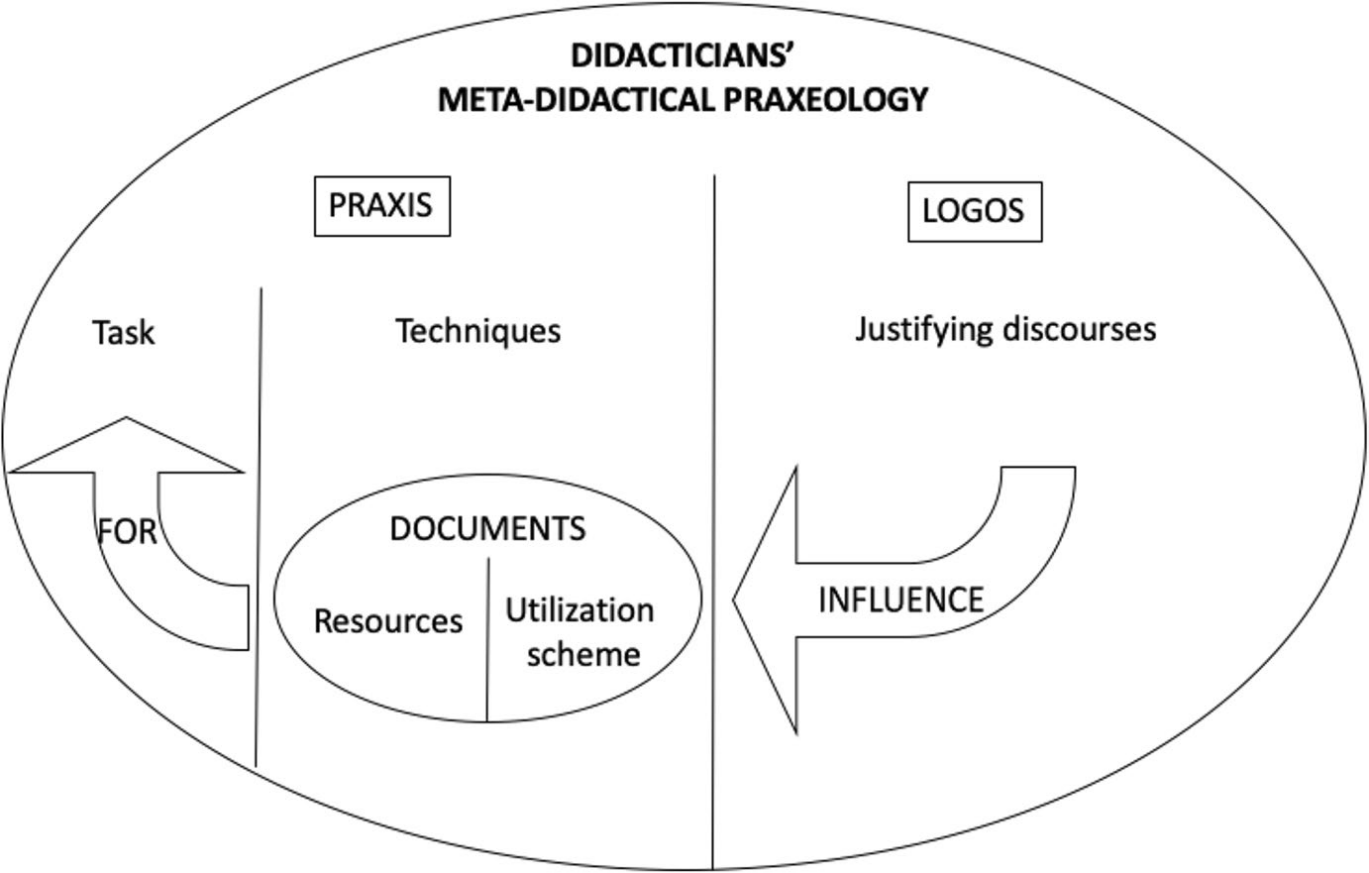
Model for the didacticians’ work, based on the MDT and the DAD frameworks

To sum up, the didacticians’ meta-didactical praxeologies referred to the design and implementation of the PD program evolved thanks to the joint work with the teachers, and this evolution is reflected in their generation of documents. Our study adds to the discussion on how didacticians’ design and implement a PD program, proposing a combined analysis with the MDT and the DAD frameworks, thus leading to a better understanding of their professional activity. In the resulting interpretative model (Fig. 5), the didacticians’ generation of documents is conceptualized as part of their techniques for the design and implementation of a PD program. The documents generated by the didacticians are intended to address the task of their meta-didactical praxeology and reflect the *logos* component. This model, generated thanks to the combination (in the sense of networking) of two well-established theoretical frameworks, the MDT and the DAD, shows the internal connections between the two frameworks. It can, therefore, contribute to improving the understanding of teachers’ PD and also provide ideas for teacher educators’ PD.

Further research could be devoted to the application of the interpretative model presented in this paper to other types of praxeologies, for example, teachers’ meta-didactical praxeologies, in relation to their documentation work. Future studies could also apply it to the analysis of PD programs led by other didacticians, different from the authors, or in the case of teacher educators who are not researchers in mathematics education.

## Data Availability

Not applicable.
